# Value-related educational goals of primary school teachers: a comparative study in two European countries

**DOI:** 10.3389/fpsyg.2024.1458393

**Published:** 2024-11-21

**Authors:** Thomas Peter Oeschger, Elena Makarova, Ella Daniel, Anna K. Döring

**Affiliations:** ^1^Institute for Educational Sciences, University of Basel, Basel, Switzerland; ^2^School of Social Sciences, University of Westminster, London, United Kingdom; ^3^Department of School Counselling and Special Education, Constantiner School of Education, Tel Aviv University, Tel Aviv, Israel

**Keywords:** value transmission, cross-sectional study, values, primary school, value-related educational goals, teachers

## Abstract

Schools serve as social institutions that convey values in the context of *socialization* and *enculturation*. Teachers are pivotal in this transmission process via their value-related educational goals (VrEGs), which outline how they would like to see their pupils in terms of values. What factors influence these VrEGs? We suggest that those vary on an individual level, but also correspond to the prevailing value orientations of the society. In our study we followed two main goals to test this thesis. Firstly, we examine the differences in VrEGs between teachers of two European countries: Switzerland (CH) and United Kingdom (UK). Secondly, we investigated the similarity of the teachers’ VrEGs with prevailing national value orientations from the specific countries. One hundred and fifty primary school teachers (108 CH, 42 UK) were asked about their VrEGs using an adapted version of Schwartz’s *Portrait Values Questionnaire* (PVQ-21). Data from the *Human Value Scale* (HVS) of the *European Social Survey* (ESS) was used for country-specific value orientations. Analyses of the value structures and the differences in value priorities showed that for the individuals from the two countries as well as for the teachers’ VrEGs from the two countries, significant differences exist in several value types. Teachers’ VrEGs in each country showed a high correlation with the corresponding national value profile. We discuss our results in light of cross-national differences in value education in schools.

## 1 Introduction

Values play a crucial role in the cohesion of a society across generations. The transmission of values to the next generation is considered one of the central socialization tasks (e.g., Roest et al., 2010; Rohan and Zanna, 1996; Schönpflug, 2001; Schwartz, 2013). Value transmission occurs through various socialization agents such as the family (e.g., Döring et al., 2017; Knafo and Schwartz, 2003; Makarova et al., 2018) the school (e.g., Berson and Oreg, 2016; Daniel et al., 2013; Luengo Kanacri et al., 2017) or peer groups (e.g., Benish-Weisman et al., 2013; Benish-Weisman, 2024).

In the processes of imparting values in education teachers occupy a central position by passing on values to pupils (Schwartz, 1992). As “socialization agents” they transmit values and norms that underlie the constitutional order (Fend, 2008). This happens on the one hand in terms of curricular educational value-related teaching objectives (VrTOs) and on the other hand in terms of what they consider important themselves to be important to their pupils in terms of values (Auer et al., 2023) by means of their value-related educational goals (VrEGs) (c.f., Oeschger et al., 2024) or socialization goals (c.f., Tamm et al., 2020).

Various studies have confirmed that countries differ in their value orientations (e.g., Cieciuch and Davidov, 2012; Hofstede, 1980, 2001; Inglehart, 1997; Schwartz, 1994, 1999; 2004). As a result, teachers’ VrEGs are likely to vary on the basis of their individual preferences, but also on the basis of correspondence with the value-related orientations prevailing in the respective country where they act as educators.

In this study, we test this hypothesis using Schwartz (1992, 1994) Value Framework to compare the country specific value orientations of individuals from Switzerland and the UK, using data from representative samples. Furthermore, we compare these country-specific value orientations with the VrEGs of primary school teachers from these two countries. In doing so, we clarify whether any differences in the teachers’ VrEGs can be linked to similarities in the differences between the specific value orientations of the two countries.

### 1.1 Values

Values express abstract motivational goals that are important to individuals in life and define what they strive for (e.g., power or security). Values influence the perceptions, attitudes, and behaviors of individuals (Sagiv and Schwartz, 2022) and are at the core of a person’s self-concept and identity (Hitlin and Piliavin, 2004). Values guide our actions by influencing our interactions with the social and natural environment and shaping its structure (Inglehart and Baker, 2000). Individuals vary in the importance they place on different values, leading to individual differences in value priorities among individuals and cultures (c.f., Fischer and Schwartz, 2011). At the same time, values are used to characterize cultural groups, and societies. Previous work confirms the occurrence of systematic cultural value differences (Hofstede, 2001; Huntington, 1993; Inglehart, 1997; Schwartz, 1999).

We used Schwartz (1992, 1994, 2012) *Theory of Basic Values* in our conceptualizations and measures, as it is well established in cross-cultural (Schwartz, 2006) as well as in educational value research (e.g., Auer et al., 2023; Berson and Oreg, 2016; Luengo Kanacri et al., 2017; Oeschger et al., 2022; Tamm et al., 2020). It therefore serves as the theoretical framework for our study and provides a solid foundation for reliable empirical research methods on values. The theory describes the main features of the structure of human values. It is considered the most widely accepted theory of values to date and has been validated in over 80 countries. The distinctive feature of this theory is that it considers different geographic, cultural, linguistic, religious, age, gender, and occupational groups (Bilsky et al., 2010; Davidov et al., 2008a, 2008b; Schwartz and Rubel, 2005).

**Figure 1:**
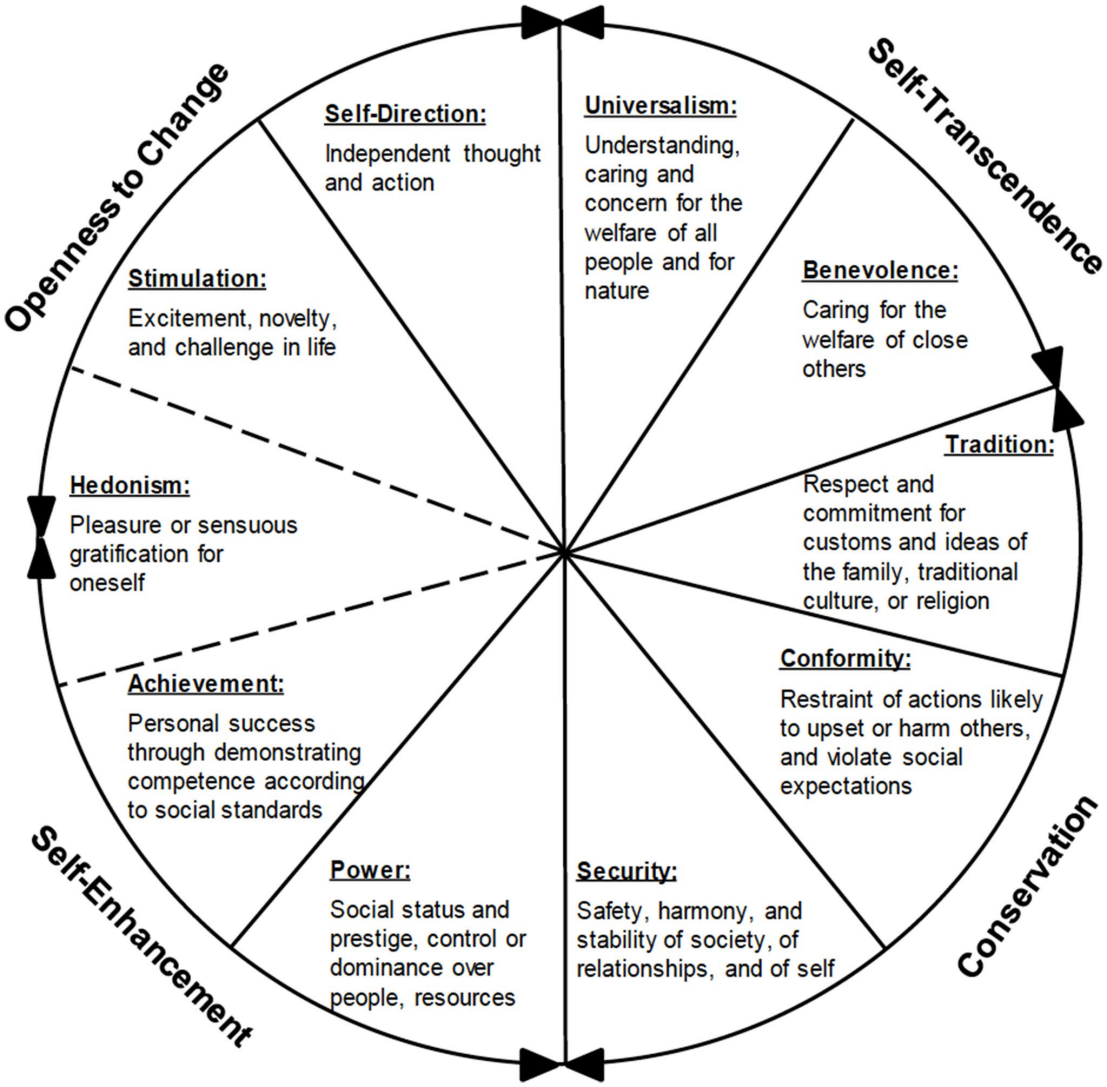
Schwartz’s theoretical model of relations among motivational types of values, higher order value types, and bipolar value dimensions (1994).

The theory suggests that single values are subsumed under 10 value types referred to as *universalism*, *benevolence*, *tradition*, *conformity*, *security*, *power*, *achievement*, *hedonism*, *stimulation*, and *self-direction* (Schwartz, 1992, 1994, 2012). The model further comprises two dimensions of opposite poles (higher order value types) which are *openness to change* (containing the value types of *self-direction*, *stimulation*, and sometimes *hedonism*; therefore, the dotted line in Figure 1) vs. *conservation* (*tradition*, *conformity*, and *security*) and *self-transcendence* (*benevolence* and *universalism*) vs. *self-enhancement* (*power* and *achievement*). For example, the pursuit of the values linked to the higher order value type of *self-transcendence* (value types of *universalism* and *benevolence*) involve the same motivation to support and help others and is compatible in this respect. However, they potentially conflict with the pursuit of the opposite values related to the higher order value type of *self-enhancement* (*power* and *achievement*), as these are aimed at gaining prestige, control, and success for oneself. On the basis of this structure, variables that relate positively to one value type tend to relate positively to neighboring value types and negatively to conflicting value types (see Figure 1). Numerous studies, such as those by Sagiv et al. (2017) or Schwartz (2003), have validated the theory, supporting the circular value type structure among individuals across different countries.

### 1.2 Value transmission in society and the school environment

The transmission of values is considered a core task of a society (Roest et al., 2010; Rohan and Zanna, 1996; Schwartz, 2013). Transmission occurs by processes of *enculturation* and *socialization* (Schönpflug, 2001), i.e., the passing of value orientations, behaviors, or attitudes (Grusec et al., 2000) to the next generation. In accordance with Bronfenbrenner (2005) ecological systems theory, value transmission is embedded in a complex system of relationships, across multiple levels of an individual’s social environment. For example, the immediate environment of the family (Döring et al., 2017; Knafo and Schwartz, 2003; Makarova et al., 2018), peers (Benish-Weisman et al., 2022; Benish-Weisman, 2024; Hurrelmann and Bründel, 2003), or the school environment (Berson and Oreg, 2016; Daniel et al., 2013; Luengo Kanacri et al., 2017); as well as the wider social environment with its general cultural values, norms, laws, and customs (Daniel et al., 2012).

Schools play an important role in the integration of children and young people into society (Standop, 2013). As social institutions, they are tasked with the reproduction of social structures (Rolff, 1997). According to Fend, this reproduction takes place by means of *enculturation and integration* as two of the four societal functions of the school system (Fend, 2008). The *enculturation* function (in the sense of cultural reproduction) aims thereby at the internalization of fundamental value orientations, by supporting students in reasoning about moral issues. The *integration* function (in the sense of preserving the internal cohesion of a society) aims at social integration through the reproduction of norms, values and world views that serve to stabilize political conditions. Both are of central significance to *value transmission* in the school environment.

Those enculturation and integration processes aim at fostering values and norms that underlie the constitutional order (Fend, 2008) and society thereby determines the legitimized values to be transferred within the educational context as policies and curricular requirements (Oeschger et al., 2022) reflecting the prevailing values of a society. These requirements are defined in terms of value-related teaching objectives (VrTOs) or value-related competences to be acquired.

### 1.3 Values education in school and teachers’ value-related educational goals (VrEGs)

The pedagogical measures that comprise the implementation of educational policy requirements take place within the framework of values education. Values education encompasses all conscious pedagogical efforts aimed at promoting and developing children’s awareness of positive values and nurturing them according to their own potential (UNESCO, 2020).

Teachers play a crucial role in this, as they act as mediators of values in the school processes of enculturation and integration. Teachers “translate” values that prevail in the broader societal context (Fend, 2008; Schwartz, 1992, 1994, 2013) by promoting students’ academic skills, emotional development, and prosocial behavior (Cadima et al., 2016) and by communicating shared social values and norms through their daily pedagogical interactions with their students in the classroom, which are guided by socially and culturally shared and accepted values (Standop, 2013).

On the one hand, these shared social values and norms exist as an educational mandate in the form of *curricular* value-related teaching objectives (Oeschger et al., 2022), the transmission of which happens consciously and intentionally through communicating expectations, modeling attitudes, structuring learning environments, encouraging through rewards, and through other classroom management practices (Wentzel and Looney, 2007).

On the other hand, values and norms are also transmitted on the basis of *extracurricular* value-related educational goals (VrEGs), which are socialization goals representing how teachers want their students to prioritize (Döring et al., 2017; Sutrop, 2015; Veugelers and Vedder, 2003). VrEGs may be transmitted through both consciously intended and unconscious unintended mechanisms, such as when teachers unconsciously act as role models (Yildirim, 2009) or when they spontaneously exemplify their value-related attitudes or behaviors through their own value-related educational goals (VrEGs).

Oeschger et al. (2024) found that teachers in the school environment convey and transmit values that are shaped by the school culture and ethos, as well as stemming from a range of curricular but also extracurricular foundations. The intentional and unintentional actions (mechanisms) by which teachers conveyed values to their students were conceptually consistent with Bardi and Goodwin's (2011) model of value change and including child-centered modeling, priming, child-led discussion, and reflection, among others (Oeschger et al., 2024).

VrEGs are moral or culturally conventional goals to which a society attaches great importance, and which have a strong connection to socially accepted virtues and moral concepts (Thornberg and Oğuz, 2016). Teachers transmit values that they consider important from a social and cultural perspective (Auer et al., 2023) and their VrEGs are considered an important element of values education in the school environment. They can be expressed by Schwartz’s 10 types of values, which have been used in previous studies (Oeschger et al., 2024; Tamm et al., 2020). Assuming that teachers’ VrEGs are based on the norms and values accepted in the social environment in which they work as educators, we expect the differences in teachers’ VrEGs between countries to be similar to the differences in the value priorities of representative samples of the respective countries.

## 2 The present studies

Currently, the authors are not aware of any studies that have examined the interplay between normative conceptions in a society and teachers’ VrEGs. Therefore, this innovative research has two main goals: (1) to examine the differences in the VrEGs between two country-specific teacher samples; (2) to investigate the similarity between VrEGs and the prevailing national value orientation. We suggest that as agents of *socialization* and *enculturation*, VrEGs of teachers are mirroring the values prevailing in their respective country. Our research analyzes data from Switzerland and the UK and is embedded in an ongoing international longitudinal research project on children’s value formation in school.^1^ A comparison of the values between the United Kingdom (UK) and Switzerland is particularly interesting because both countries represent two areas in Schwartz’s cultural values map for Western Europe and the Anglo-Saxon countries (Schwartz, 2008).

*Study 1* compares the country-specific value orientations of Switzerland and the UK based on the 10 *value types* according to Schwartz (1992, 1994) by means of representative samples of the two countries from the *Human Value Scale* (HVS) of the *European Social Survey* (European Social Survey European Research Infrastructure, 2023) to display possible differences in value orientations of individuals from the two countries. *Study 2* compares teachers’ VrEGs based on the same values by means of two samples of primary school teachers from the same two countries.

Both studies follow the same methodology. To answer the question of the similarity in value priorities between the respective country sample and the corresponding teachers’ VrEGs, we additionally analyzed the similarity between the value-related national profiles with the VrEGs of the teachers from the respective country.

### 2.1 Study 1—differences in value orientations between Switzerland and the United Kingdom

As a first step, we aimed to identify the value priorities prevalent in two representative samples of individuals from Switzerland and the UK.

#### 2.1.1 Methods

##### 2.1.1.1 Sample

The Swiss sample in our study included *N* = 1,523 participants (48.6% female). The age of the participants ranged from 15 to 90 years, with *M_age_* = 49.59 (*SD* = 18.86). The UK sample in our study included *N* = 1,149 participants (55% female). The age of the participants ranged from 15 to 90 years, with a *M_age_* = 55.71 (*SD* = 18.29). Both samples are representative of the population of specific country (European Social Survey European Research Infrastructure, 2023).

##### 2.1.1.2 Instrument

We used the *Human Values Scale* (*HVS*) of the *ESS* which is a well-established and widely used 21-item value measure developed by Schwartz et al. (2015). The scale is based on *Schwartz’ Portrait-Value-Questionnaire* (*PVQ-21*, 1994) and consists of 21 items that include a brief verbal portrait describing a person’s life goals or aspirations. For each item, respondents indicate how similar the person described in the description is to themselves using a 6-point Likert scale (from 6 = “not at all like me” to 1 = “very much like me”). Two (or three for the *value type* of *universalism*) items represent each of the 10 value types defined by Schwartz. The *PVQ-21* is an adequate instrument allowing the mean comparison of the postulated Schwartz values for cross-cultural research accounting for invariance properties of the scales used (Cieciuch and Davidov, 2012).

##### 2.1.1.3 Procedure of data collection and data processing

Data for this study was taken from *Round 10* (September 20–22) of the *ESS* which contains overall data from 31 countries. For our studies’ purposes we only analyzed data from the Swiss and the UK sample. We centered the rating of the 21 value items from the *HVS* on the respective mean of all items for each participant to correct for individual differences in use of the response scale. This procedure for centering has become a common procedure in value research (Bardi et al., 2014). It is also suitable for eliminating multicollinearity between individual data and context characteristics and for reducing covariances between regression coefficients and constants. SPSS (Version 27.0.1.0), was used as data processing software.

##### 2.1.1.4 Analysis using ESS data from the representative Swiss and UK sample

For the analysis of value structures in two *ESS* datasets from Switzerland and the UK, we implemented a theory-guided ordinal *multidimensional scaling* (MDS) (Borg et al., 2018) technique, to depict a two-dimensional layout of relationships among value types derived from the *PVQ-21*. This technique maps the correlations between the importance assigned to value items, into spatial distances within a two-dimensional space. Strong correlations, indicating that participants who highly rate a particular value item, are also likely to highly rate another, result in these items being placed close to each other in this spatial arrangement. This proximity occurs especially when these items share similar correlation patterns with all other items. To enhance our analysis toward a more accurate representation, we utilized a theoretical framework as an initial setup, following recommendations by Borg and Mair (2017). We further applied an *unfolding analysis* (Borg et al., 2017, 2018) to determine the individual-level structure of values, a method recently used in values research (Barni and Knafo-Noam, 2012; Daniel et al., 2013), originating from Coombs (1964) unfolding theory of preferential choice. This approach allowed us to construct a model for each person’s hierarchy of values, exploring if individuals show preference for values that are theoretically aligned in a similar manner and have distinct preferences for values that are theoretically opposed. These analyses were performed using the *smacof* package (Version 2.1-5) in R (de Leeuw and Mair, 2009), and the adequacy of the model fit to the data was evaluated through permutation tests and assessing the congruence of individuals and values with the model (Mair et al., 2016). To investigate the country specific differences in the value orientations between the two *ESS* samples, we used independent means *t*-test (independent measures *t*-test) to analyze the aggregated means for each value type from the *HVS* for the two groups (Switzerland/UK). SPSS (Version 27.0.1.0) was used as analytic software.

#### 2.1.2 Results

All descriptive and correlative analysis for the *HVS data* is reported in the supplementary material, in Supplementary Table S1 (for all 21 items) and Supplementary Table S2 (aggregated to 10 value types) for the Swiss sample and in Supplementary Table S3 (for all 21 items) and Supplementary Table S4 (aggregated to 10 value types) for the UK sample.

##### 2.1.2.1 Swiss and UK ESS sample value structure

**Figure 2:**
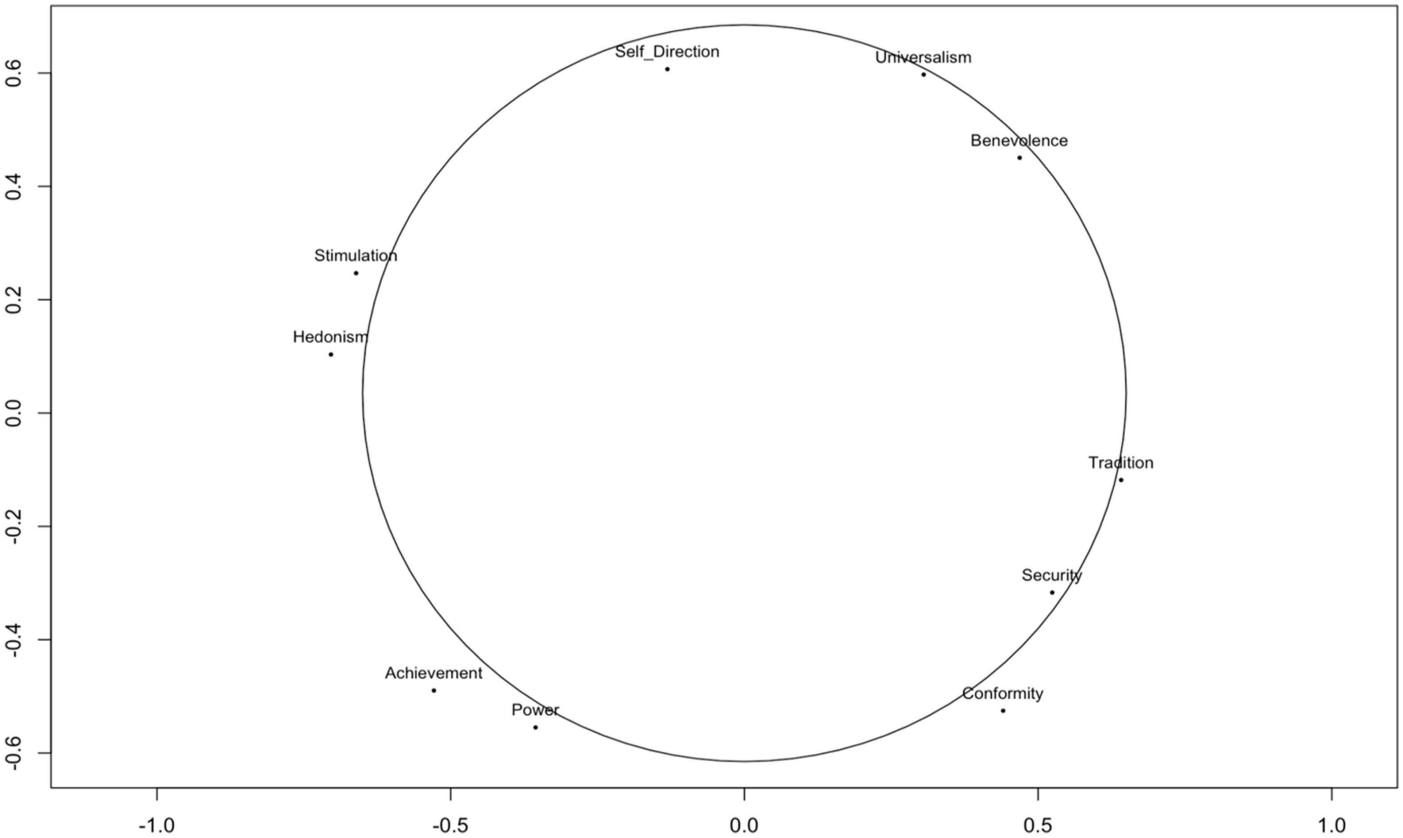
Value structure for the Swiss and the UK ESS sample. Sample size (*N* = 1,523 CH, *N* = 1,149 UK). Using all 21 items from the *HVS* aggregated to Schwartz’s 10 value types.

The estimated *MDS* model fits our data from the two *ESS* samples well (*Stress-1* = 0.056) according to the criteria for *MDS* model fit (Spence and Ogilvie, 1973). Figure 2 indicates that our results show the circular structure as hypothesized by Schwartz’ theory.^2^

##### 2.1.2.2 Unfolding of the intraindividual value structure using ESS data from the Swiss and the UK ESS sample

**Figure 3:**
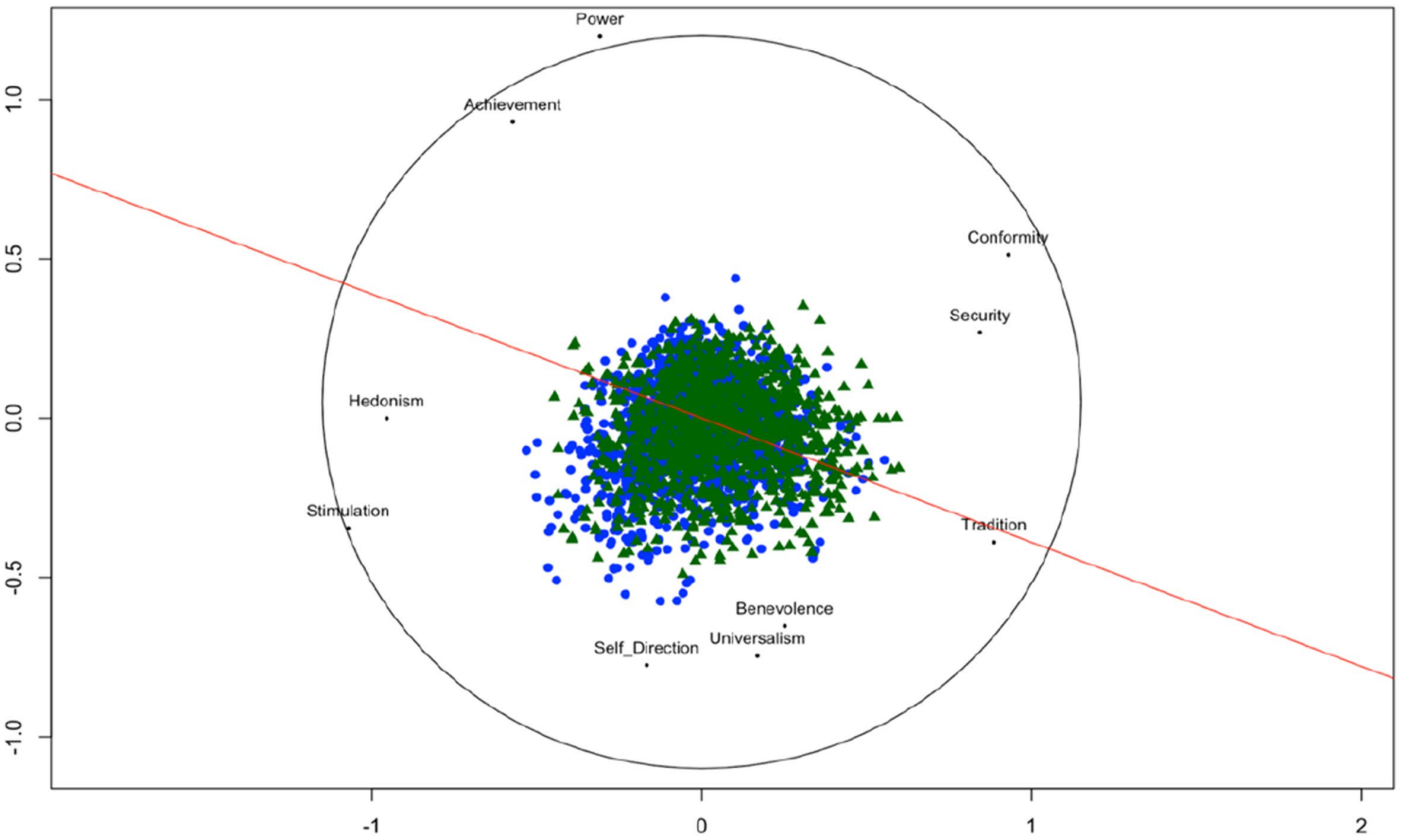
Unfolding solution for personal values from ESS data for the UK and the Swiss sample. Sample size (*N* = 1,523 CH, *N* = 1,149 UK). Using all 21 items from the *HVS* aggregated to Schwartz’s 10 value types.

The *unfolding* solution of each individual and their value preferences revealed the expected circular structure of all 10 *value types* following *Schwartz’s Value Framework*. Figure 3 displays each person from the two *ESS* samples (UK/CH) in a joint space such that the distances between each person’s point and each object point (value type) represent the observed preference value type as green triangles (UK) and blue dots (CH). The arrangement of the 10 *value types* corresponds to the circular theoretical structure with all values at their corresponding place in the correct sector according to Schwartz’ theory (see text footnote 2 respectively). The *Stress-1* value of our unfolding solution is 0.185 which indicates a good fit according to Mair et al. (2016) and is significantly lower (average permutation stress = 0.26, *p* < 0.01) based on a 100 permutations test (Borg et al., 2018). The red line represents the discriminant for origin (UK/CH). This is the line on which persons from UK and from Switzerland are best separated [*t*(2191.9) = −10.491, *p* < 0.01]. Viewed along the red discriminant line individuals from the UK (green triangles) tend to lie significantly more at the value types of *tradition*, *conformity,* and *security* end of this scale, where individuals from Switzerland (blue dots) tend to lie closer to the value types of *hedonism* and *stimulation* and *self-direction*.

The solid red line is the discriminant line for origin (c.f., Borg et al., 2018). Green triangles represent UK individuals, blue tringles represent individuals from Switzerland.

##### 2.1.2.3 Differences in value orientations from Switzerland and the United Kingdom

The independent two sample *t*-test showed significant differences in the means for value types of *universalism*, *benevolence*, *conformity*, *security*, *power*, *hedonism,* and *self-direction* between the Swiss and the UK *ESS* sample. The Swiss *ESS* sample significantly valued the value types of *hedonism, power,* and *self-direction* more than the UK *ESS* sample. The UK *ESS* sample significantly valued the value types of *universalism*, *benevolence*, *conformity,* and *security,* more than the Swiss *ESS* sample. Effect sizes ranged from small to middle size according to Cohen (1988). All results are reported in Table 1.

**Table 1:** *T*-test results comparing the Swiss and the UK ESS sample (21-items HVS) on Schwartz’s 10 value types.

	CH		UK		*Cohen’s d*	*t*	*df*
	*M*	*SD*	*M*	*SD*			
Universalism (UN1, UN2, UN3)	0.61	0.64	0.82	0.68	−0.32	−8.11**	2,662
Benevolence (BE1, BE2)	0.88	0.58	0.98	0.64	−0.15	−3.86**	2,659
Tradition (TR1, TR2)	0.02	0.85	0.02	0.91	−0.01	−0.08	2,655
Conformity (CO1, CO2)	−0.46	0.92	−0.30	1.05	−0.17	−4.29**	2,657
Security (SE1, SE2)	0.07	0.83	0.44	0.87	−0.43	−10.89**	2,650
Power (PO1, PO2)	−1.00	0.83	−1.25	0.84	0.30	7.63**	2,658
Achievement (AC1, AC2)	−0.60	0.94	−0.65	1.00	0.05	1.33	2,656
Hedonism (HE1, HE2)	0.15	0.80	−0.40	0.91	0.65	16.60**	2,654
Stimulation (ST1, ST2)	−0.64	0.97	−0.62	1.04	−0.18	−0.47	2,654
Self-direction (SD1, SD2)	0.62	0.69	0.55	0.82	−0.10	2.59*	2,658

Data is centered and aggregated to 10 value types. *p < 0.05, **p < 0.01.

#### 2.1.3 Summary

The Results of *Study 1* revealed on the one hand that our data followed the intended theoretical structure according to *Schwartz’s Value Framework*. On the second hand the unfolding of all individuals from the two countries showed that there is a significant difference in value orientations between the two samples analyzed. Further, the independent two sample *t*-test showed significant differences for specific *value types* between the Swiss and the UK *ESS* sample.

VrEGs are theoretically suggested to be based on socially legitimized norms and ideas prevailing and accepted in a society (Veugelers and Vedder, 2003). Following the differences in value orientations between the representative *ESS* samples from Switzerland and the UK found in *Study 1*, we predicted that the teachers’ VrEGs from the two countries would similarly differ. The following *Study 2* aimed to test this hypothesis.

### 2.2 Study 2—differences in value-related educational goals of primary school teachers from Switzerland and the United Kingdom

In *Study 2*, we investigated the hierarchy of VrEGs for two teacher samples, from Switzerland and the UK. Furthermore, in a second step, we investigated whether these respective value types differ significantly between the two samples examined. Last, we investigated the similarity between the values of the teacher samples and the national samples introduced in *Study 1*.

#### 2.2.1 Methods

##### 2.2.1.1 Sample

The Swiss teacher sample in our study included 108 primary school teachers (93.3% female) teaching year 1 and 2 of Primary School in Switzerland (D-EDK, 2016) recruited with the permission of the respective education departments from public primary schools in seven cantons of the German-speaking part of Switzerland. The age of the sample ranged from 21 to 64 years with a *M_age_* = 38.33 (*SD* = 13.04). In all, 104 teachers (96.3%) were born in Switzerland with 14 (3.7%) in another country. Their average teaching experience was 12.8 years (range 1–39 years, *SD* = 11.2). The UK teacher sample included 42 primary school teachers (88.5% female) teaching in *Key Stages 1* and *Key Stage 2* recruited with the permission of the school headmasters from 11 schools from South England areas (Berkshire, Surrey, Greater London, and Essex). The age of the sample ranged from 23 to 63 years with a *M_age_* = 41.4 (*SD* = 12.67). In all, 35 teachers (83.3%) were born in the UK with 7 (16.7%) in another country. Their average teaching experience was *M_exp_* = 10.4 years (range 0–35 years, *SD* = 8.31).

##### 2.2.1.2 Instrument

We adapted Schwartz’ *PVQ-21* to assess teachers’ VrEGs. The *PVQ-21* was described in Study 1. In our study, we adapted it to assess VrEGs, which are defined as the values that teachers want to see in their pupils. This operationalization was already applied and validated among parents (Döring et al., 2017). Participants were presented with the following question: “Imagine that the pupils in your class would fill in this questionnaire. How would you like your pupils to complete it? It is not about what the children are really like, but about what answers you would like them to give. How similar do you want your pupils to be to the people described?” Teachers rated how much they wanted their pupils to resemble the person described in each of the 21 portraits by using a 6-point Likert scale (from 1 = “not at all like them” to 6 = “very much like them”).

##### 2.2.1.3 Procedure of data collection and data processing

The participating teachers of both samples provided data on their VrEGs in Spring 2022. Unipark’s EFS Survey tool (version 22.2) was used for the online surveys and the individual survey links were emailed to the teachers 1 week before the survey was launched. The study was approved by the ethical committee of the (anonymized for submission). All data collected was pseudonymized to not allow any conclusions to be drawn about the respective individuals. We centered the rating scores of the 21 value items from the VrEGs on the respective mean of all items for each participant to correct for individual differences in use of the response scale (MRAT) in the same way as in *Study 1*.

##### 2.2.1.4 Analysis using teachers’ VrEGs data from the Swiss and UK teacher sample

We conducted the same theory-guided ordinal *multidimensional scaling* (MDS) and *unfolding* techniques (Borg et al., 2017, 2018) and *t*-test procedure for the analysis of the VrEGs in the two teachers’ datasets from Switzerland and the UK as described in *Study 1*.

##### 2.2.1.5 Analysis of the similarity of teachers VrEGs with the national profiles

To measure the extent to which each teachers’ value profile reflected his or her national profiles, dyadic correlations were utilized. This method, also known as q-correlation, involves calculating the Pearson product–moment correlation for two groups of scores within each pair, as described by Kenny and Winquist (2001). Applied to values, it allows to analyze the degree to which individuals agree in terms of the relative importance they give to a broad range of values, in our case to the value importance in the country. Dyadic correlations range from −1 to +1 where positive correlations indicate that individuals are similar in terms of the profiles of their ratings, whereas negative correlations suggest the opposite (Barni et al., 2014). According to Cohen (1988) coefficients lower than 0.30 indicate a small, coefficients between 0.30 and 0.50 a moderate, and coefficients higher than 0.50 indicate a large agreement. To calculate the agreement with the corresponding national profile in our study, we correlated each teachers’ value profile with his or her average national profile. Correlations were then transformed using Fisher’s *r* to *Z* transformation to conduct significance tests of mean correlations: Indeed, the resulting values have a standard normal or *Z* distribution under the null hypothesis that the average correlations are 0. The *r* to *Z* transformation provides exact tests and confidence intervals for comparing two (or more) correlations (Malloy and Albright, 2001).

This procedure of analysis of the agreement with individuals and their national profiles by calculating correlation between two value profiles has already been applied in previous research for individuals (Barni et al., 2014) as well as for parents’ value-related educational goals (Döring et al., 2017). In order to provide further insight into the results, the teachers’ VrEGs were correlated with the opposite national value profile. This was done in order to ascertain whether the correlations differed significantly from those observed in the origin countries.

#### 2.2.2 Results

All descriptive and correlative analysis for the VrEGs is reported in the supplementary material Supplementary Table S5 (for all 21 items) and Supplementary Table S6 (aggregated to 10 value types) for the Swiss teachers’ sample and in Supplementary Table S7 (for all 21 items) and Supplementary Table S8 (aggregated to 10 value types) for the UK teachers’ sample.

##### 2.2.2.1 Swiss and UK teachers’ VrEGs structure

**Figure 4:**
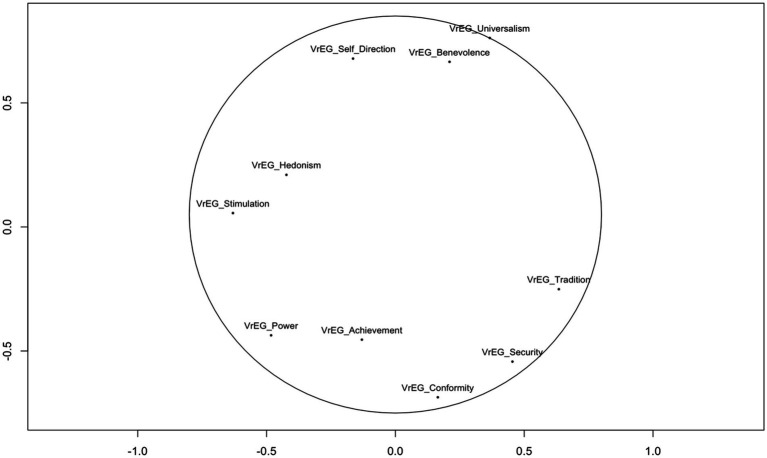
Value structure for the teachers’ VrEGs for the UK and the Swiss sample. Sample size (*N* = 108 CH, *N* = 42 UK). Using all 21 items from the PVQ-21 aggregated to Schwartz’s 10 value types.

The estimated *MDS* model fits our data from the two samples of teachers’ VrEGs well (*Stress-1* = 0.129) according to the criteria for a *MDS* model fit (Spence and Ogilvie, 1973). The graphical illustration in Figure 4 indicates that our results show some minor deviations of the position of the values but follow the circular structure as foreseen by the theoretical structure according to Schwartz’ *Value Framework* (see text footnote 1 respectively).

##### 2.2.2.2 Unfolding of the intraindividual value structure using data from the Swiss and UK teachers’ VrEGs sample

**Figure 5:**
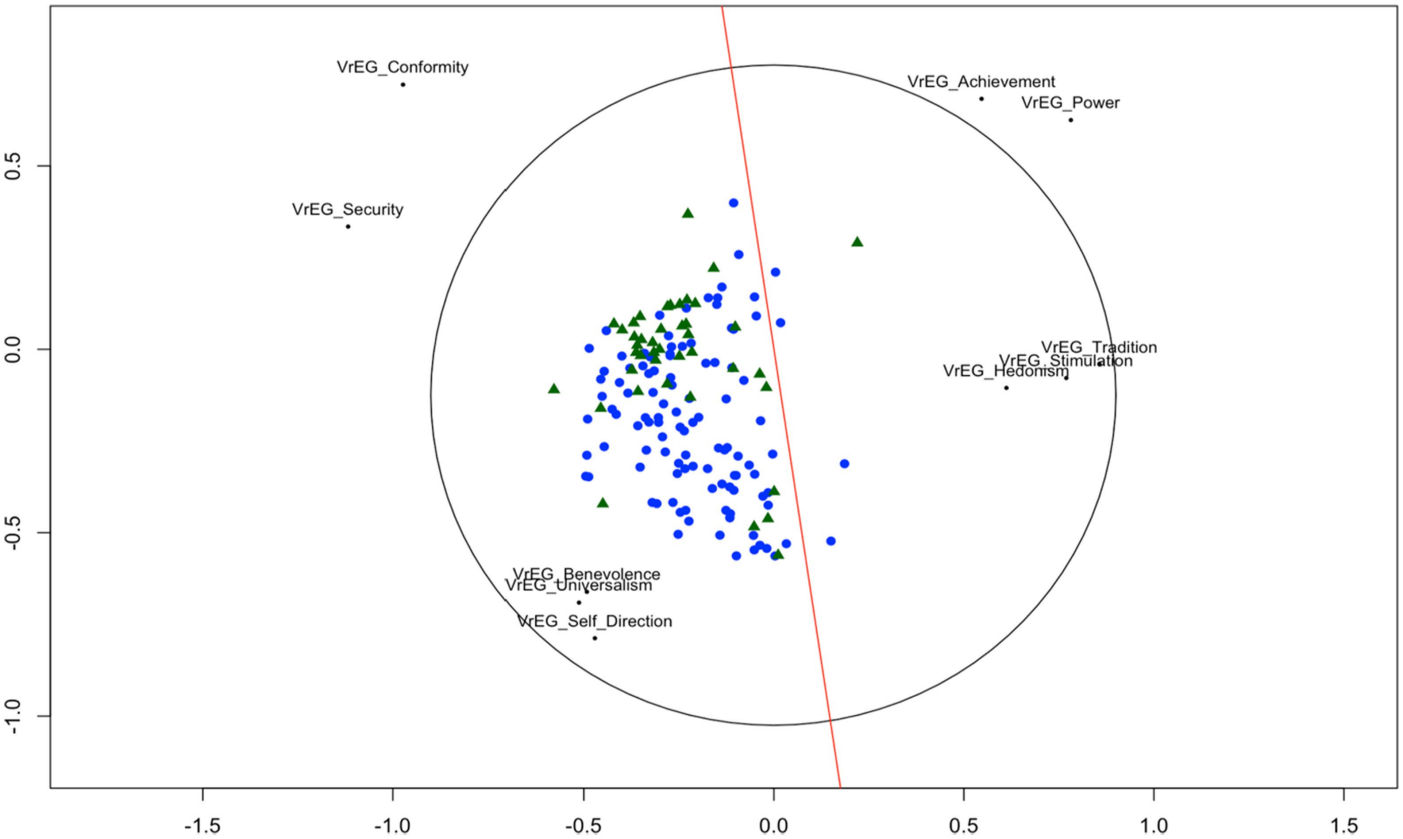
Unfolding solution for 10 value types from teachers’ VrEGs data for the UK and the Swiss teachers’ samples. Note. Sample size (*N* = 108 CH, N = 42 UK). Using all 21 items from the PVQ-21 aggregated to Schwartz’s 10 value types.

The unfolding solution of each teacher and their value preferences in their VrEGs revealed the expected circular structure of all 10 *value types* following *Schwartz’s Value Framework*. Figure 5 displays each teacher from the two samples in a joint space such that the distances between each teacher’s point and each object point (value type) represent the observed preference value type as green triangles (UK) and blue dots (CH). The *Stress-1* value of the solution is 0.22, which indicates a good fit according to Mair et al. (2016) and is significantly lower (average permutation stress = 0.29, *p* < 0.01) based on a 100 permutations test (Borg et al., 2018). The red line represents the discriminant line for origin (UK/CH). This is the line on which teachers from UK and from Switzerland are best separated [*t*(79.941) = −4.1154, *p* < 0.01]. Teachers from the UK (green triangles) tend to lie more at the value types of *conformity*, *security, power, and achievement* end of this scale, where teachers from Switzerland tend to lie closer to *self-direction*, *universalism,* and *benevolence* with their VrEGs.

The solid red line is the discriminant line for origin (c.f. Borg et al., 2018). Green triangles represent UK teachers, blue tringles represent teachers from Switzerland.

The *unfolding* solution of each teacher and their value preferences in their VrEGs revealed the expected circular structure of all 10 value types following *Schwartz’s Value Framework* except for the value type of *tradition* which is not correctly located at its corresponding place according to Schwartz’s theory. As a result, we repeated the unfolding procedure again without the value type of *tradition*.

**Figure 6:**
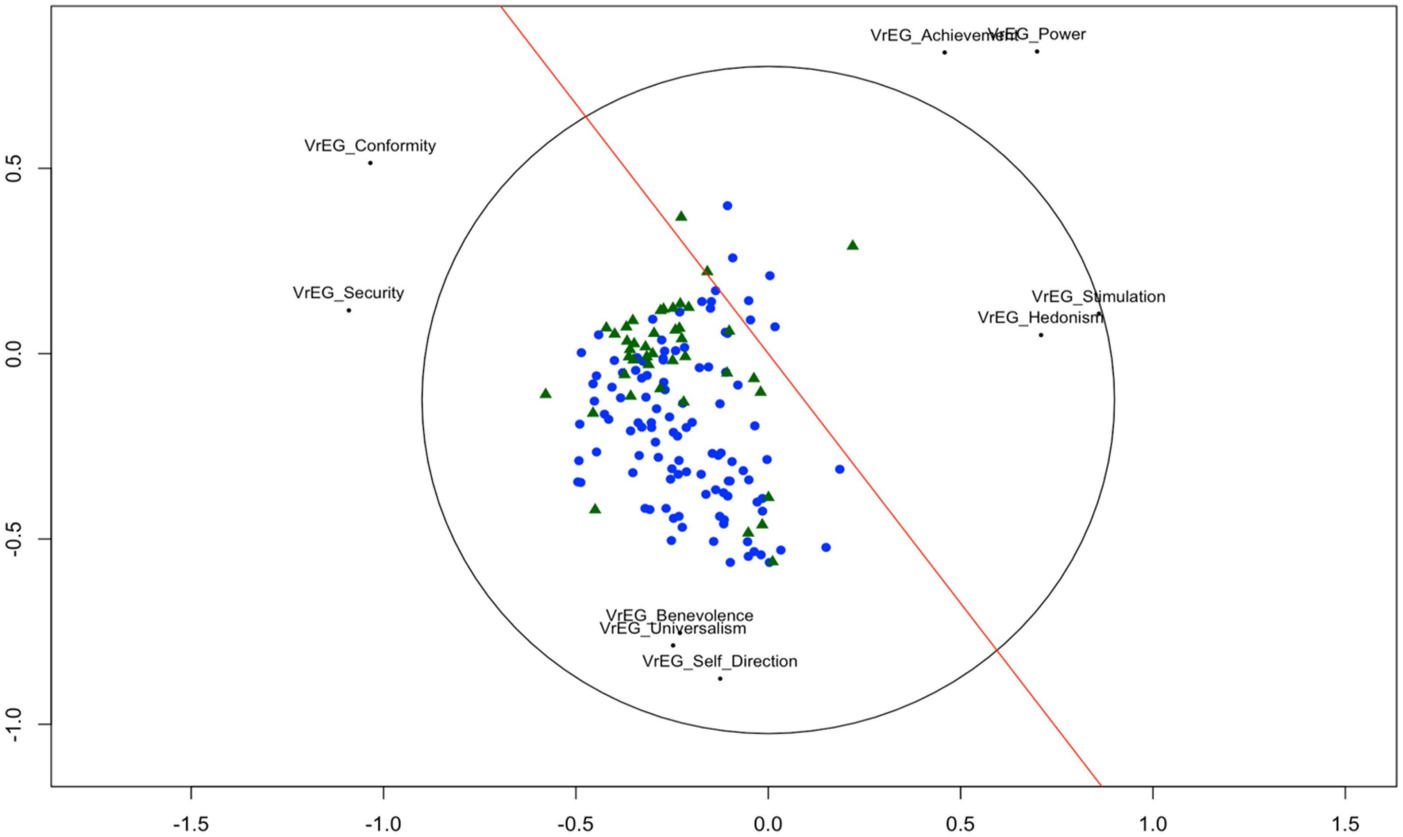
Unfolding solution for nine value types (without tradition) from teachers’ VrEGs data for the UK and the Swiss teachers’ sample. Sample size (*N* = 108 CH, *N* = 42 UK). Using all 21 items from the PVQ-21 aggregated to Schwartz’s 10 value types.

Figure 6 displays each teacher’s VrEGs from the two teachers’ samples as green triangles (UK) and blue dots (CH). The unfolding of each teacher’s individual VrEGs in relation to the whole sample without the value type of *tradition* revealed now the expected circular value structure. The arrangement of the values corresponds now to the circular theoretical structure with all values at their corresponding place. *The Stress-1* value of the solution is 0.21, which indicates a good fit according to Mair et al. (2016) and is significantly lower (average permutation stress = 0.29, *p* < 0.01) based on a 100 permutations test (Borg et al., 2018). The red line represents the discriminant line for origin (UK/CH). This is the line on which teachers from UK and from Switzerland are best separated [*t*(77.051) = −5.0336, *p* < 0.01]. Teachers from the UK (green triangles) tend to lie more at the value types of *conformity,* and *security* end of this scale, where teachers from Switzerland (blue dots) tend to lie closer to *self-direction*, *universalism,* and *benevolence* with their VrEGs.

The solid red line is the discriminant line for origin. Green triangles represent UK teachers, blue tringles represent teachers from Switzerland.

##### 2.2.2.3 Differences in teachers’ VrEGs from Switzerland and the UK

The independent two sample *t*-test showed significant differences in the means of the value types of *benevolence, tradition, conformity, security, achievement, hedonism, stimulation,* and *self-direction* between the VrEGs of the Swiss and the UK Teachers’ sample. Effect sizes ranged from middle to large size according to Cohen (1988) except for value types of *universalism* and *power*. All results are reported in Table 2.

**Table 2:** *T*-test results comparing the Swiss and the UK teachers’ VrEGs (21-items from adapted PVQ-21) on Schwartz’s 10 value types.

	Switzerland		UK		*Cohens’s d*	*t*	*df*
	*M*	*SD*	*M*	*SD*			
Universalism (UN1, UN2, UN3)	1.03	0.80	0.81	0.81	0.27	1.49	148
Benevolence (BE1, BE2)	1.22	0.64	0.67	0.73	0.83	4.59**	148
Tradition (TR1, TR2)	−0.66	1.01	−0.18	0.82	−0.51	−2.79*	148
Conformity (CO1, CO2)	−0.82	1.02	−0.35	1.04	−0.46	−2.52*	148
Security (SE1, SE2)	−0.42	0.96	0.05	0.98	−0.49	−2.69*	148
Power (PO1, PO2)	−1.05	0.89	−1.20	1.01	0.17	0.91	148
Achievement (AC1, AC2)	−0.83	1.04	−0.24	1.00	−0.57	−3.16**	148
Hedonism (HE1, HE2)	0.49	0.78	−0.39	0.86	1.09	5.99**	148
Stimulation (ST1, ST2)	−0.35	0.81	0.01	0.68	−0.46	−2.55*	148
Self-direction (SD1, SD2)	0.88	0.68	0.41	0.54	0.74	4.05**	148

Data is centered and aggregated to 10 value types. *p < 0.05, **p < 0.01 (two-sided).

In addition to looking at the differences and effect sizes, we also calculated the *coefficient of overlap (OVL)* and the absolute effect to show the extent to which the two distributions of the VrEGs of teachers from the UK and Switzerland overlap for each value type and to show the average differences in the scale points of the two groups for their VrEGs. Hanel et al. (2019) emphasize the advantages of this method, especially in cross-cultural research, to achieve more balanced scientific communication by presenting similarity information. The degree of overlap measured by the OVL - regardless of its statistical significance - provides information about the “practical relevance” of the difference in the VrEGs of the teachers from the two countries. Teachers’ VrEGs are most similar (most overlapping) with regard to the value types of power (OVL = 0.89; which represents an overlap of 89%) and universalism (OVL = 0.94; which represents an overlap of 94%). This confirms the results of the non-significant results of the *t*-test and the effect sizes (*d* = 0.27 for universalism and *d* = 0.17 for power) for these two value types. All results are shown in Supplementary Table S9.

##### 2.2.2.4 Value similarity between teachers’ VrEGs and the national profiles

The Swiss teachers’ VrEGs have an average correlation of *r* = 0.67 (*p* < 0.01) with the Swiss country *ESS* values profile, which indicates a large effect size according to Cohen (1988). To check, the results were also correlated with *ESS* data from the other country. The Swiss teachers’ VrEGs have an average correlation of *r* = 0.58 (*p* < 0.01) with the British country *ESS* values profile. A paired *t*-test showed that the correlation of the Swiss teachers’ VrEGs with the Swiss country *ESS* values profile is significantly higher than the correlation with the British country *ESS* values profile [*t*(107) = 11.54, *p* < 0.01] (one-sided).

The British teachers’ VrEGs have an average correlation of *r* = 0.60 (*p* < 0.01) with the British country *ESS* values profile, which also indicates a large effect size according to Cohen (1988). To check, the results were also correlated with *ESS* data from the other country. The British teachers’ VrEGs have an average correlation of *r* = 0.55 (*p* < 0.01) with the Swiss country *ESS* values profile. A paired *t*-test showed that the correlation of the British teachers’ VrEGs with the British country values profile is significantly higher than the correlation with the Swiss country *ESS* values profile [*t*(41) = 3.02, *p* < 0.01] (one-sided).

#### 2.2.3 Summary

The Results of *Study 2* revealed that the 21 items from the VrEGs follow the intended theoretical structure according to *Schwartz’s Value Framework* except for the value type *tradition*. The unfolding of all teachers in both samples showed that there is a significant difference between the two samples analyzed and that teachers from the UK (green triangles) tend to lie more with their VrEGs at the value types of *conformity* and *security* end of this scale, where teachers from Switzerland (blue dots) tend to lie closer to *self-direction*, *universalism,* and *benevolence* with their VrEGs.

In line with the hypothesis that the teachers’ VrEGs reflect the country-specific value orientation, we found similar patterns of prioritization for the four value types of *conformity*, *security*, *hedonism,* and *self-direction*. Table 3 provides a summarized overview of the results under this perspective.

**Table 3:** Overview of the directions of the means of the corresponding value types between the ESS sample (Study 1) and the teachers’ VrEGs (Study 2).

Value type (Items from PVQ-21)	HVS from ESS		Teachers’ VrEGs	
	*UK*	*CH*	*UK*	*CH*
Universalism (UN1, UN2, UN3)	+	−	n.s.	n.s.
Benevolence (BE1, BE2)	+	−	−	+
Tradition (TR1, TR2)	n.s.	n.s.	+	−
**Conformity (CO1, CO2)**	**+**	**−**	**+**	**−**
**Security (SE1, SE2)**	**+**	**−**	**+**	**−**
Power (PO1_PO2)	−	+	n.s.	n.s.
Achievement (AC1, AC2)	n.s.	n.s.	+	−
**Hedonism (HE1, HE2)**	**−**	**+**	**−**	**+**
Stimulation (ST1, ST2)	n.s.	n.s.	+	−
**Self-direction (SD1, SD2)**	**−**	**+**	**−**	**+**

Those value types that show the same directional pattern for the corresponding ESS and the VrEGs are marked in bold. + Indicates that the mean for this value type is higher in the specific sample compared to the other sample. −Indicates that the mean for this value type is lower in the specific sample compared to the other sample. n.s., not significant.

## 3 Discussion

Educational goals possess a normative nature and serve as a means to ensure a society’s self-preservation and cultural continuity (Brezinka, 1990, 1995). Since VrEGs are constituted by the values and norms prevailing in a society (Veugelers and Vedder, 2003), we hypothesized differences between specific countries when it comes to what educators - in our case teachers - consider important for their pupils in terms of values (VrEGs) and similarity between teachers’ VrEGs and country-specific value orientations (national profiles) based on their country of origin.

Emphasizing the role of values in teachers’ thinking and practice (Court, 1991) the structural analysis of the VrEGs of teachers revealed that the inter-relations between values resembled the hypothesized structure. These results suggest that the teachers in our samples, in their view of the ideal pupil, follow the structure of individual values. They do not aspire for pupils to embrace all values but make trade-offs between conflicting values. For example, they desire their pupils with their VrEGs to either follow rules closely (“*They believe that people should do what they are told. They think people should follow rules at all times, even when no one is watching”;* Item_CO_01) or be highly creative (“*They like surprises and are always looking for new things to do. They think it is important to do lots of different things in life”;* Item_ST_01); to support their friends (“*It is important to them to be loyal to their friends. They want to devote themselves to people close to them”;* Item_BE_02) or excel and lead (“*It is important to them to get respect from others. They want people to do what they say”;* Item PO_02). All 21 items representing the teachers’ VrEGs can be found in the Supplementary Data Sheet 1. Marušić-Jablanović (2018) and Pudelko and Boon (2014) thereby highlight the importance of understanding teachers’ values in shaping their classroom goals and expectations for students. These findings underscore the need for teachers to recognize and make trade-offs between conflicting values, as suggested by the analysis. However, some deviations were found in the structure of educational values, more at the individual than at the sample level. These deviations suggest that the value type *tradition* did not adhere to the circle of values closely. The religious affiliation of schools is a strong external constrain on school values (Hofmann-Towfigh, 2007), and shapes the school norms and climate. As a result, teachers may be less likely to shape their educational goals independently, in line with their other values. The Data shows a better structure without accounting for the two items measuring the value type of *tradition* in our adapted *PVQ-21*. Item 1 for the value type of *tradition* in the *HVS* reads: “*It is important to them to be humble and modest. They try not to draw attention to themselves.”* This statement emphasizes an individual, inner attitude (modesty and restraint), which manifests itself in the personal way of life and in dealing with others. Item 2 reads: “*Tradition is important to them. They try to follow the customs handed down by their religion or their family.”* This statement on the other hand, emphasizes the importance of external, overarching structures (traditions and customs) that are brought to the individual from outside and shape their way of life. We suspect that even though both views reflect important aspects of human values, teachers and their VrEGs they focus on different areas of social and personal life.

When examining the similarities between the VrEGs and values in national profiles of the *ESS* samples — specifically where the mean values of value priorities are significantly higher or lower in both *ESS* and VrEGs samples — we find the same directional patterns: Value types of *conformity* and *security* are higher in the UK than in Switzerland, while value types of *hedonism* and *self-direction* are higher in Switzerland than in the UK (Table 3). Moreover, the evidence found of the similarity of teachers‘VrEGs to corresponding national values profiles (0.59 for UK and 0.67 for Switzerland) underpins the involvement of teachers’ VrEGs when it comes to the transmission of values and norms underpinning the constitutional order, and supports Fend (2008) theory that the school environment - and in our case teachers in particular—promotes the integration of the next generation into society by transmitting the values and norms reflecting the constitutional order. This is consistent with findings that suggest that educational goals of educators reflect what is prevalent and accepted in a society (Döring et al., 2017; Sutrop, 2015; Veugelers and Vedder, 2003). Based on the VrEGs/ESS directional patterns, the differences can now be discussed from different perspectives with regard to the school environment.

The timing of our survey (*ESS* Round 10 from September to September 22) and VrEGs (early spring 2022) largely took place during or shortly after the COVID-19 pandemic phases in Switzerland (from March 2020 to February 2022; Oxenius and Karrer, 2022) and the UK (from March 2020 to December 2021; Institute for Government, 2022). Firstly, it is notable that, at the time of the survey around the pandemic, British teachers placed greater importance on the value types of *conformity* and *security* than their counterparts in Switzerland. This was evident in their responses regarding the importance of following rules, keeping others safe, and caring for vulnerable people through some safety behaviors. However, British teachers also placed greater importance on following rules for their students in terms of preventive growth than their Swiss counterparts. As Tan et al. (2020) and Knolle et al. (2021) show, the UK was more affected by the COVID-19 crisis than any other European country. The pandemic situation at the time of our surveys may therefore have affected the value priorities of individuals in the UK (both teachers and *ESS* sample) in terms of the types of values that represent caring for vulnerable people by engaging in certain safety behaviors, but also following rules or ensuring the safety of others. Work by Daniel et al. (2021) or Sneddon et al. (2022) supports this assumption by showing that specific temporal contexts (such as a pandemic) can cause a change in individuals’ value orientations. Although the COVID measures also affected Swiss schools, they were less noticeable in Switzerland than in the UK and there were far fewer prolonged school closures in Switzerland than in the UK, as confirmed by the *OECD* report The State of Global Education (OECD, 2021).

Secondly, examining the directional patterns for the value types of *hedonism* and *self-direction* needs a closer look at the curricula from both countries, as they reflect society’s mandate to the school and provide a possible explanation. A curriculum represents the educationally legitimized mandate of society as a formulated educational mission for schools and conveys values, that are considered significant by a society (Scheunpflug et al., 2024). The value-related content set out in it aims to pass on the values that apply in society to the next generation via the school. As a result, it can be assumed that, as shown, it is not only the value-related educational goals of teachers as a means of transmitting social values that differ between countries, but also the value-related content of the curriculum. Several studies deal with curriculum research and values but focus primarily on analyzing specific content such as the anchoring of global learning topics in the curricula of different countries (Schreiber and Siege, 2016; Killick, 2020). A comparative analysis of values-based curriculum content between different countries does not yet exist. Oeschger et al. (2022) pave the way as the first by analyzing the Swiss Curriculum (Lehrplan 21, D-EDK, 2016) for the first 2 years of Kindergarten and primary school. In this curriculum, almost 40% of the implicit values mentioned could be assigned to the *value types* of *self-direction* and *hedonism*.

Similar studies analyzing Schwartz’s 10 *value types* rooted in the UK curriculum do not exist. However, one can examine the *Fundamental British Values* (FBV) introduced in Department of Education (2014) as part of the British counter-terrorism policy, as a strategy to support the ‘Prevent Duty’ (British Government, 2023) to deter pupils from extremism (Lockley-Scott, 2019). These *FBVs* include democracy, the rule of law, individual liberty and mutual respect and tolerance of people with different faiths and beliefs (British Government, 2021, 2023). At first glance, these *FBVs* can be linked to Schwartz’s *value types*, with democracy and the rule of law corresponding to the value type of *universalism*, individual freedom to *self-direction* and mutual respect and tolerance to *benevolence* and *universalism*. However, it should be noted that these links are not direct and may vary according to individual interpretation and cultural context. Such varied interpretation has led to criticism of the promotion of the *FBV* in the national curriculum: as conflicting with universal human rights (Struthers, 2017) perpetuating a sense of ‘us and them’ and alienation, particularly for foreign citizens (c.f., Crawford, 2017; Lander, 2016) or even reinforcing a racialized social order underpinned by nationalist, colonial values (Winter, 2018). This criticism therefore implies that *FBVs* can also be understood as value types of *conformity* and *security* according to Schwartz (1994) and in particular teachers must be aware of this ambiguity.

### 3.1 Limitations

Given the non-experimental nature of our research, we cannot rule out the possibility that additional factors influence the obtained findings of our study. For example, the sample of teachers surveyed in our study may be too small to draw generally valid conclusions and, due to the recruitment carried out as part of the (anonymized for submission) research project, may not be representative of the overall population of teachers. These factors could limit the generalisability of the results. In addition, the pandemic situation during our surveys could have had an impact on the relevance of teachers’ value-related educational goals, especially in terms of the value types of *security* and *conformity*. The fact that specific temporal contexts can bring about a change in the value orientations of individuals (Daniel et al., 2021; Sneddon et al., 2022) or teachers’ value-related educational goals (Oeschger et al., 2024) has already been demonstrated.

Davidov et al. (2008a, 2008b) tested the cross-national measurement invariance of the PVQ-21 several times with data from the European Social Survey (ESS) and pointed out the problem that in most of the countries analyzed, only seven values could be identified at the configuration level, which made it necessary to combine some pairs of adjacent values (such as *power* with *achievement*, *benevolence* with *universalism* and *conformity* with *tradition*) (Davidov et al., 2008a, 2008b). However, Cieciuch et al. (2014) note that this does not contradict Schwartz’s theory, as neighboring values express similar motivations, and any division of the continuum is to some extent arbitrary.

Although Schwarz’s work confirms the occurrence of similar cultural regions and thus the existence of systematic cultural value differences as shown in several previous empirical studies (Hofstede, 2001; Inglehart, 1997; Huntington, 1993) any reference to these findings to explain generally differences in value orientations under a cultural perspective must however be treated with caution due to the *ecological fallacy* that has to be taken into account when comparing values on an individual and an cultural level (Brewer and Venaik, 2014).

### 3.2 Implications

Regarding values education in schools, some important implications can be drawn from our results for the school environment, for teacher education but also for supranational consideration.

*Strengthening the relevance of value education:* The uncovered alignment between the values in a society and the VrEGs of teachers suggests that the two education systems we studied are well positioned to prepare pupils for their lives in society. If the values transmitted in school are in line with those of society, this increases the relevance of education for pupils’ personal and professional development. Further on this consensus influences also political and social discussions about the role of education in society and emphasizes the importance of education as a place that not only imparts knowledge, but also contributes to shaping social and cultural cohesion.

*Facilitating the transmission of values for teachers:* Teachers may find it easier to teach values that resonate both with what is important to them for their pupils in the classroom and in wider society. This could contribute to more effective value education and social integration of pupils.

*The need and benefit for critical reflection on values: A*lthough a high degree of congruence between the values prevalent in society and teachers VrEGs offers offer advantages, it is important that pupils critically reflect on the values they are taught. Schools should offer a space in which pupils learn to critically question social norms and values and develop their own convictions. National Curricula such as the *Swiss National Curriculum* already take this into account by explicitly formulating competencies that require pupils to be able to explain, examine and defend their own values and norms (D-EDK, 2016). Teachers, on the other hand, must be made aware - especially in the context of teacher education and training - that their own values and their value-related educational goals match to a specific extent (Tamm et al., 2020). During their professional activities as educators, teachers’ value-related educational goals express the extent to which teachers want their pupils to adopt the values underlying a social norm. These values could be expressed in everyday actions and behaviors and may not necessarily align with curricular value-related teaching objectives (VrTOs). It is essential that teachers, as the most important agents of socialization in the school system, acquire a reflective sensitivity for the manifestations of values develop an awareness of the interactions between their personal values, the curricular VrTOs and their VrEGs in course of their training and professionalization.

*Adaptability:* The correlation between the values prevailing in a society and the teachers’ VrEGs also shows that the education system and its stakeholders must be able to react sensitively to societal changes. Values and norms continue to evolve as we have seen, e.g., during the pandemic, and the education system should be able to adapt to these changes to remain relevant. The similarity shown in our study between national value profiles and teachers’ VrEGs sheds light on how closely education and society are linked in terms of values. This highlights the need for educational institutions to continually engage with society, striking a balance between teaching societal values and promoting critical thinking and diversity.

### 3.3 Outlook and further directions

In order to develop an even deeper understanding of the relationship between value orientations and teachers’ VrEGs, further comparative studies could be carried out in other national contexts. This would make it possible to check the generalizability of the results found for other countries, and to obtain a broader picture of the interdependencies. Longitudinal studies on changes in societal value orientation would further be useful to investigate how value orientations change over time and how this influences teachers’ VrEGs. The same study design applied to teachers from other school levels would also provide valuable insights into the level of deviation between teachers’ VrEGs and social conventions across education levels. And finally, an investigation of how educational policy decisions influence the value orientation in schools and teachers VrEGs could provide valuable insights into the interactions between policy making and value education.

## Data Availability

The raw data supporting the conclusions of this article will be made available by the authors, without undue reservation.

## Footnotes

^1^Anonymized for submission.

^2^Minor swaps in positioning are possible and are discussed in detail in Schwartz (1992) work.
