# Supplementary material for: Value-related educational goals of primary school teachers: a comparative study in two European countries
Source: Front Psychol. 2024 Nov 21;15:1458393. doi: 10.3389/fpsyg.2024.1458393 (PMC11619050; doi:10.3389/fpsyg.2024.1458393)
Supplement: Supplementary file 1 [file Data_Sheet_1.pdf]

Table S10

21-item Questionnaire for the teachers' value-related educational goals adapted from the PVQ-21 (Schwartz, 1994)

Question: "*Imagine that the pupils in your class would fill in this questionnaire. How would you like your pupils to complete it? It is not about what the children are really like, but about what answers you would like them to give. How similar do you want your pupils to be to the people described?"*

Item	Wording
PVQ_UN1	They think it is important that every person in the world be treated equally. They believe everyone should have equal opportunities in life.
PVQ_UN2	It is important to them to listen to people who are different from them. Even when they disagree with them, they still want to understand them.
PVQ_UN3	They strongly believe that people should care for nature. Looking after the environment is important to them.
PVQ_BE1	It's very important to them to help the people around them. They want to care for their well-being.
PVQ_BE2	It is important to them to be loyal to their friends. They want to devote themselves to people close to them.
PVQ_TR1	It is important to them to be humble and modest. They try not to draw attention to themselves.
PVQ_TR2	Tradition is important to them. They try to follow the customs handed down by their religion or their family.
PVQ_CO1	They believe that people should do what they're told. They think people should follow rules at all times, even when no-one is watching.
PVQ_CO2	It is important to them always to behave properly. They want to avoid doing anything people would say is wrong.
PVQ_SE1	It is important to them to live in secure surroundings. They avoid anything that might endanger their safety.
PVQ_SE2	It is important to them that the government ensures their safety against all threats. They want the state to be strong so it can defend its citizens.
PVQ_PO1	It is important to them to be rich. They want to have a lot of money and expensive things.
PVQ_PO2	It is important to them to get respect from others. They want people to do what they say.
PVQ_AC1	It's very important to them to show their abilities. They want people to admire what they do.
PVQ_AC2	Being very successful is important to them. They hope people will recognise their achievements.
PVQ_HE1	Having a good time is important to them. They like to "spoil" themselves.
PVQ_HE2	They seek every chance to have fun. It is important to them to do things that give them pleasure.
PVQ_ST1	They like surprises and are always looking for new things to do. They think it is important to do lots of different things in life.
PVQ_ST2	They look for adventures and like to take risks. They want to have an exciting life.
PVQ_SD1	Thinking up new ideas and being creative is important to them. They like to do things in their own original way.
PVQ_SD2	It is important to them to make their own decisions about what they do. They like to be free and not depend on others.

Coding: [1] not like them at all, [2], not like them, [3] a little like them, [4] somewhat like them, [5] like them, [6] very much like them.
