# Supplementary figures and images for: Value-related educational goals of primary school teachers: a comparative study in two European countries
Source: Front Psychol. 2024 Nov 21;15:1458393. doi: 10.3389/fpsyg.2024.1458393 (PMC11619050; doi:10.3389/fpsyg.2024.1458393)

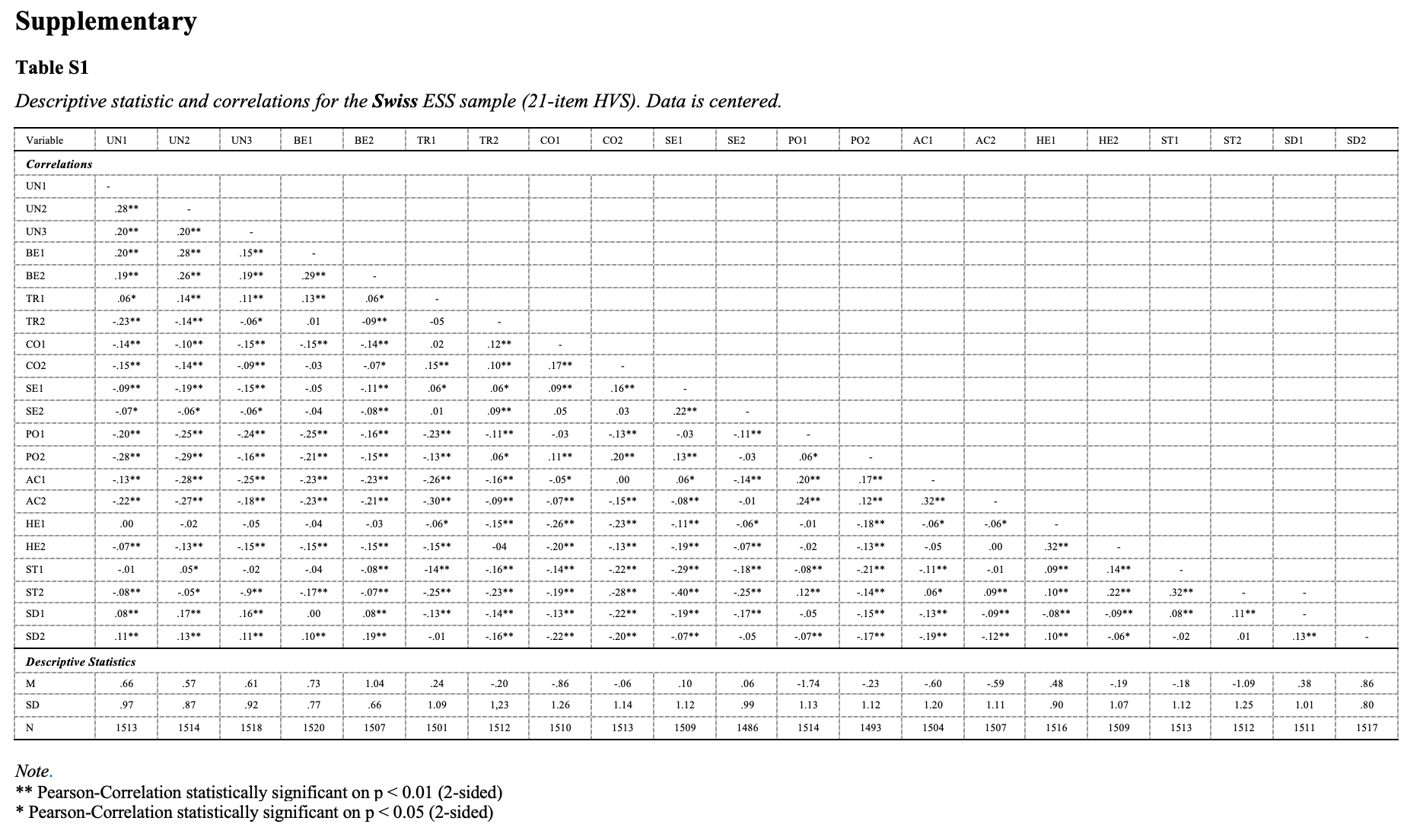

Supplement: Supplementary file 2 [file Image_1.png]

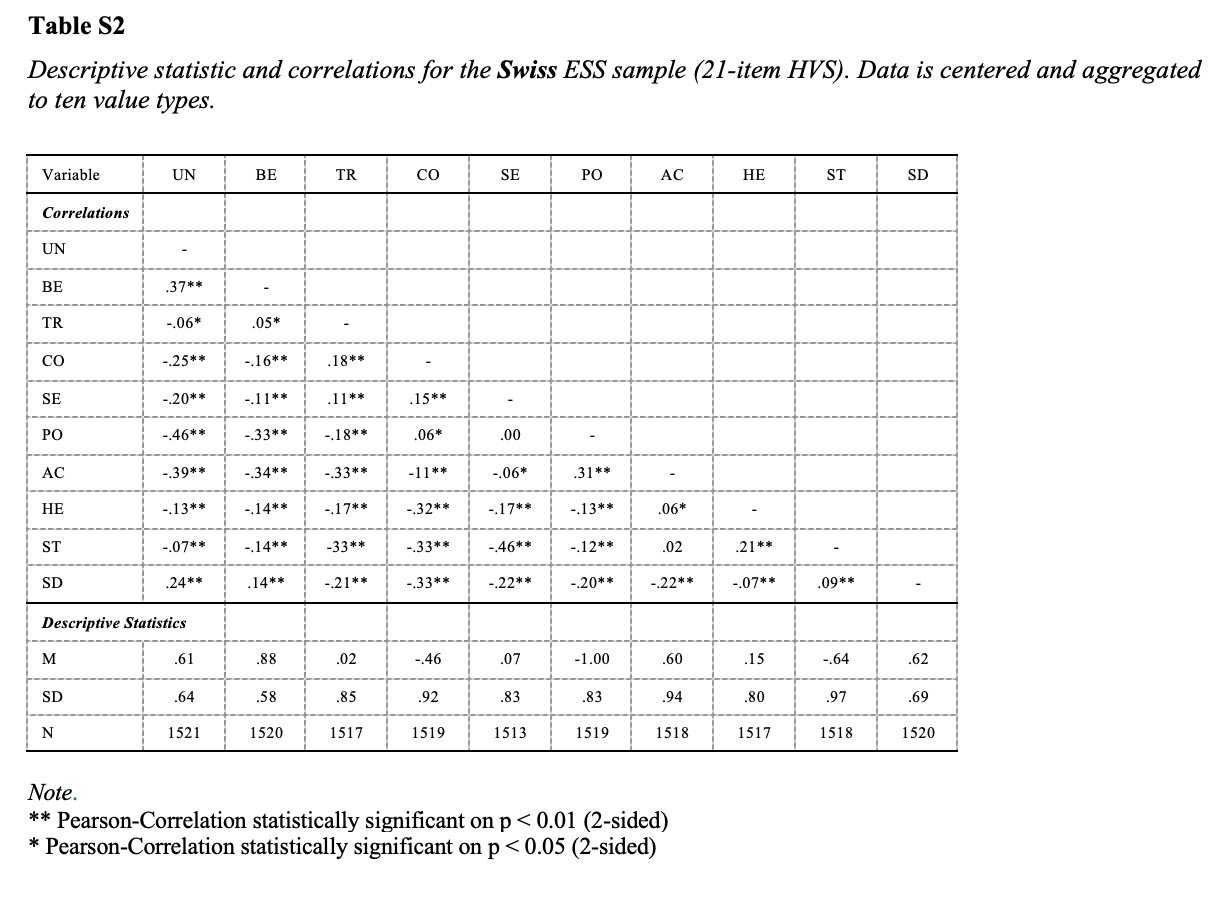

Supplement: Supplementary file 3 [file Image_2.png]

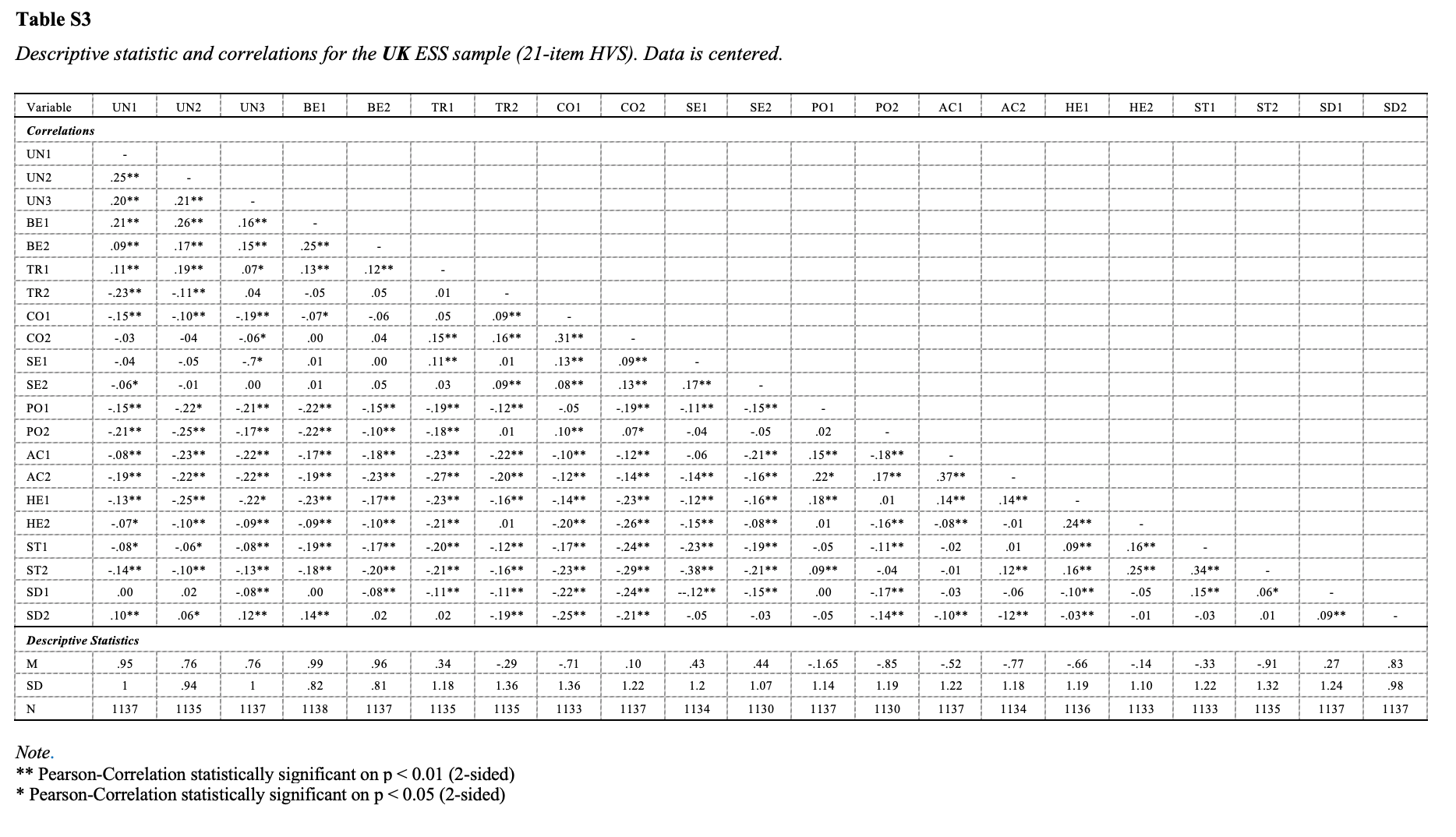

Supplement: Supplementary file 4 [file Image_3.png]

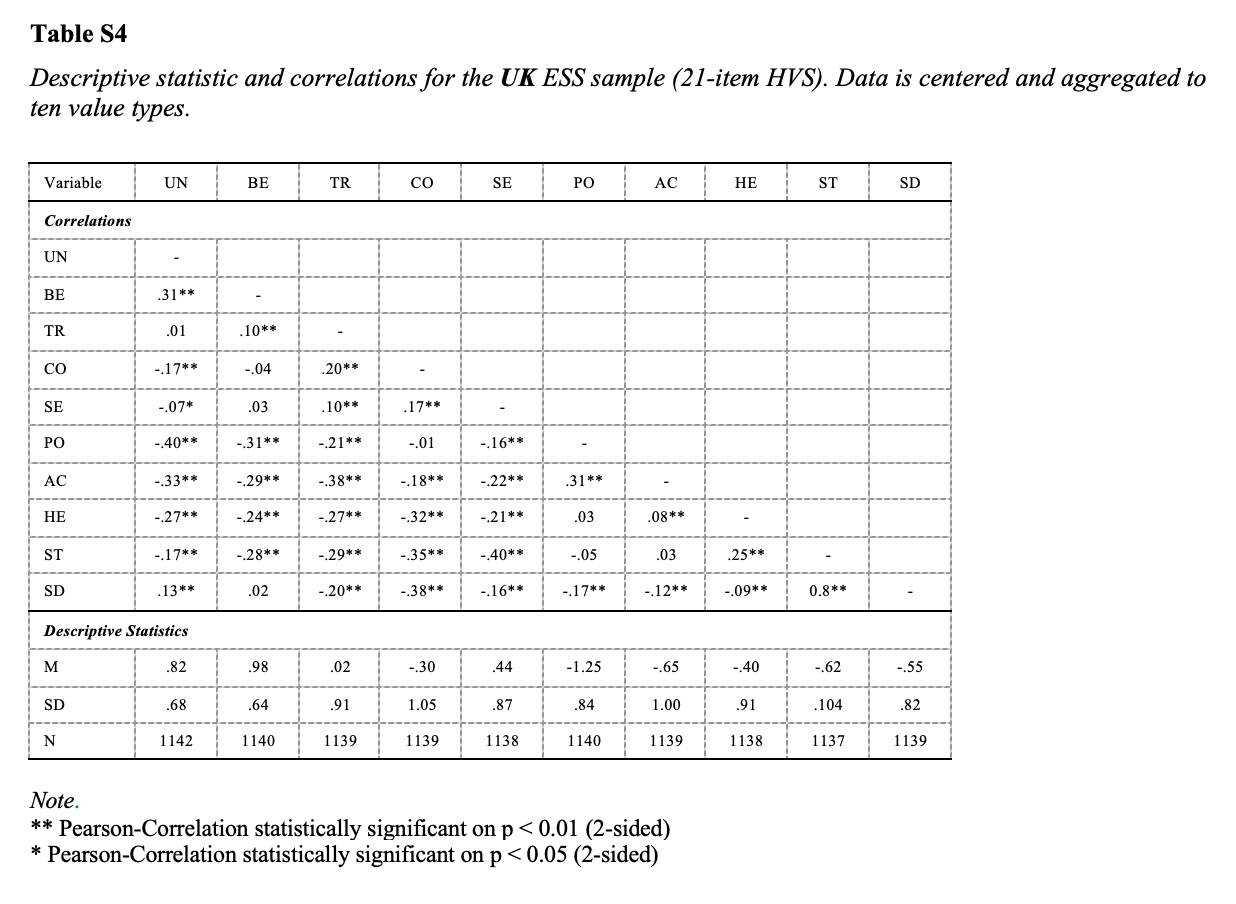

Supplement: Supplementary file 5 [file Image_4.png]

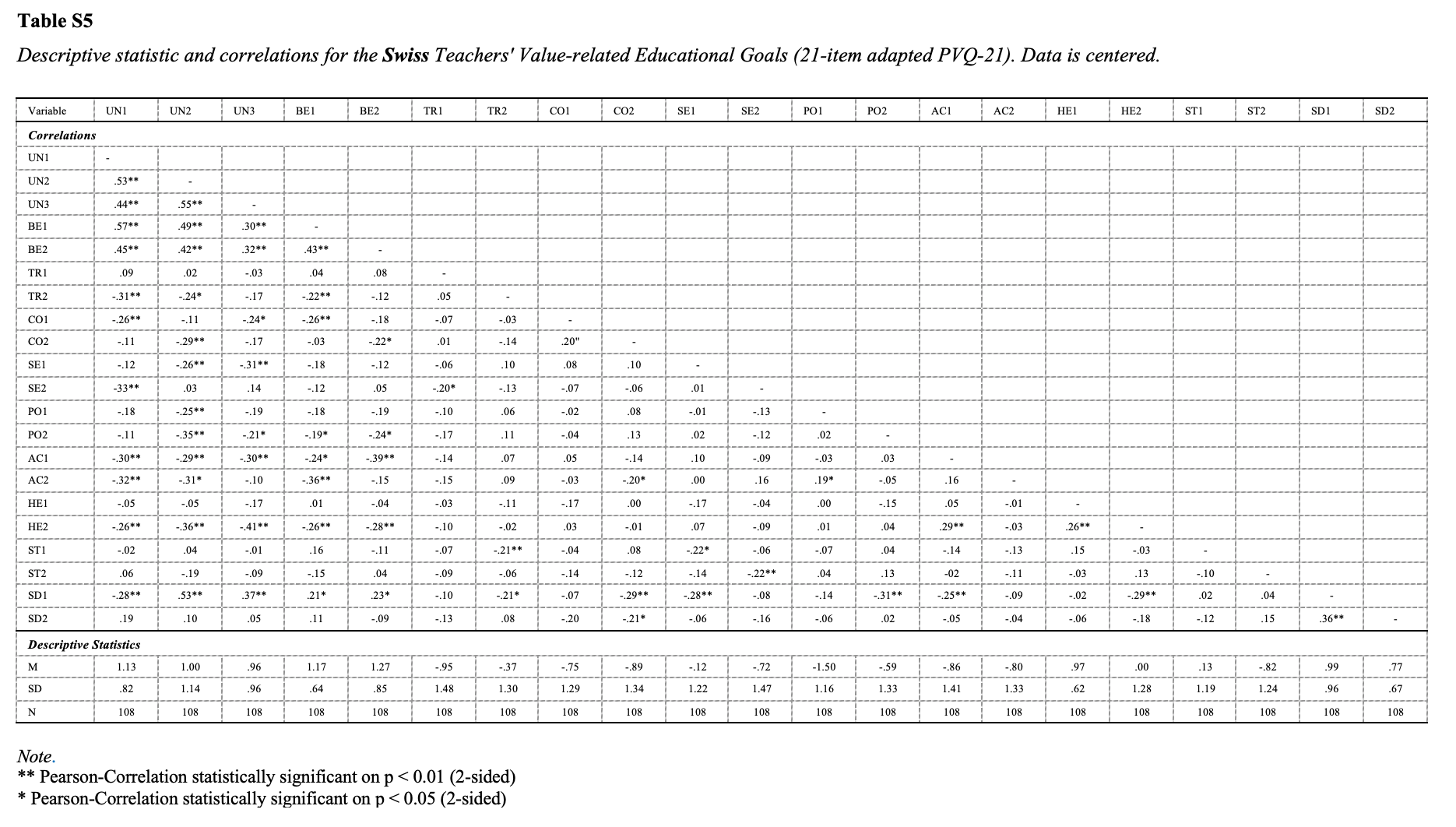

Supplement: Supplementary file 6 [file Image_5.png]

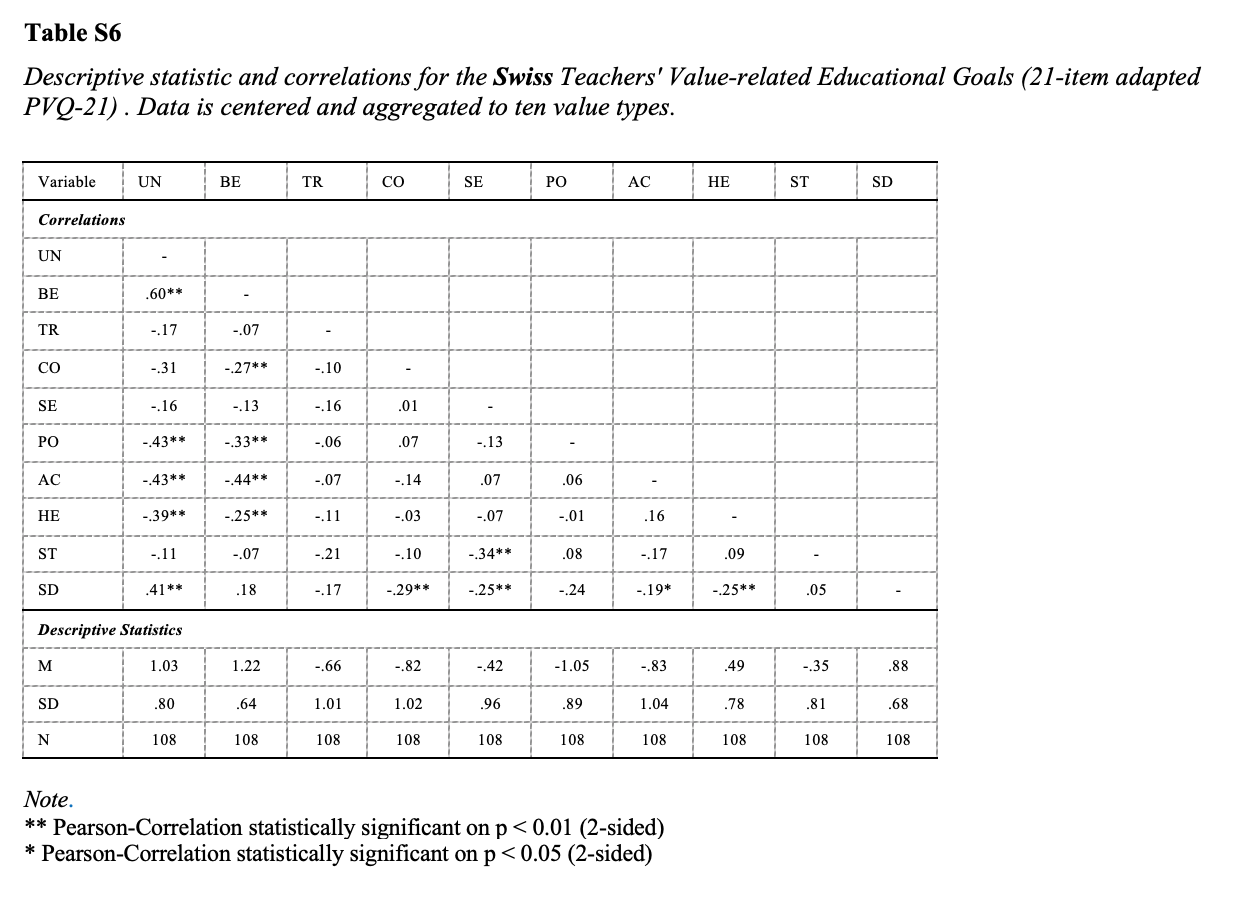

Supplement: Supplementary file 7 [file Image_6.png]

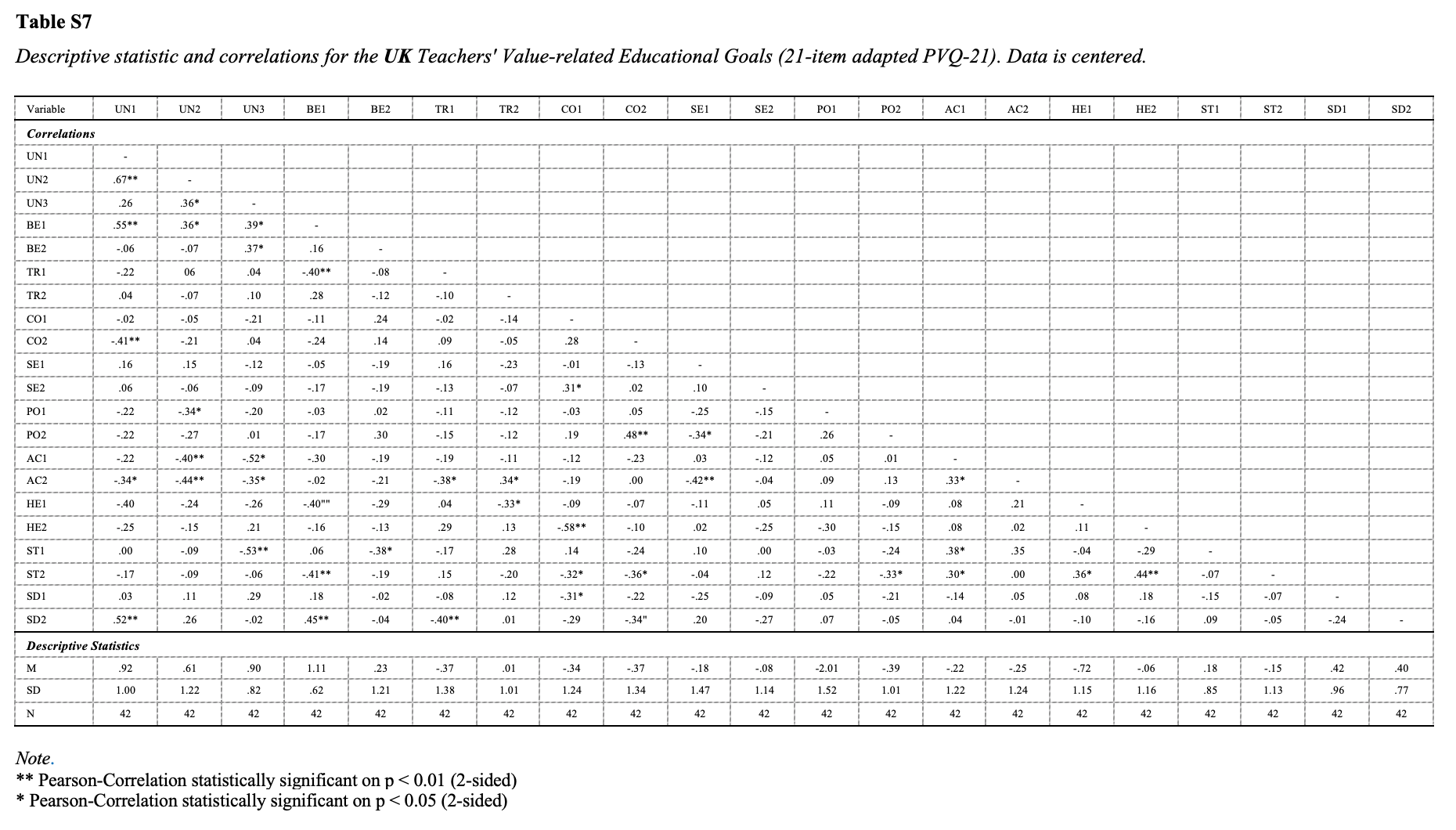

Supplement: Supplementary file 8 [file Image_7.png]

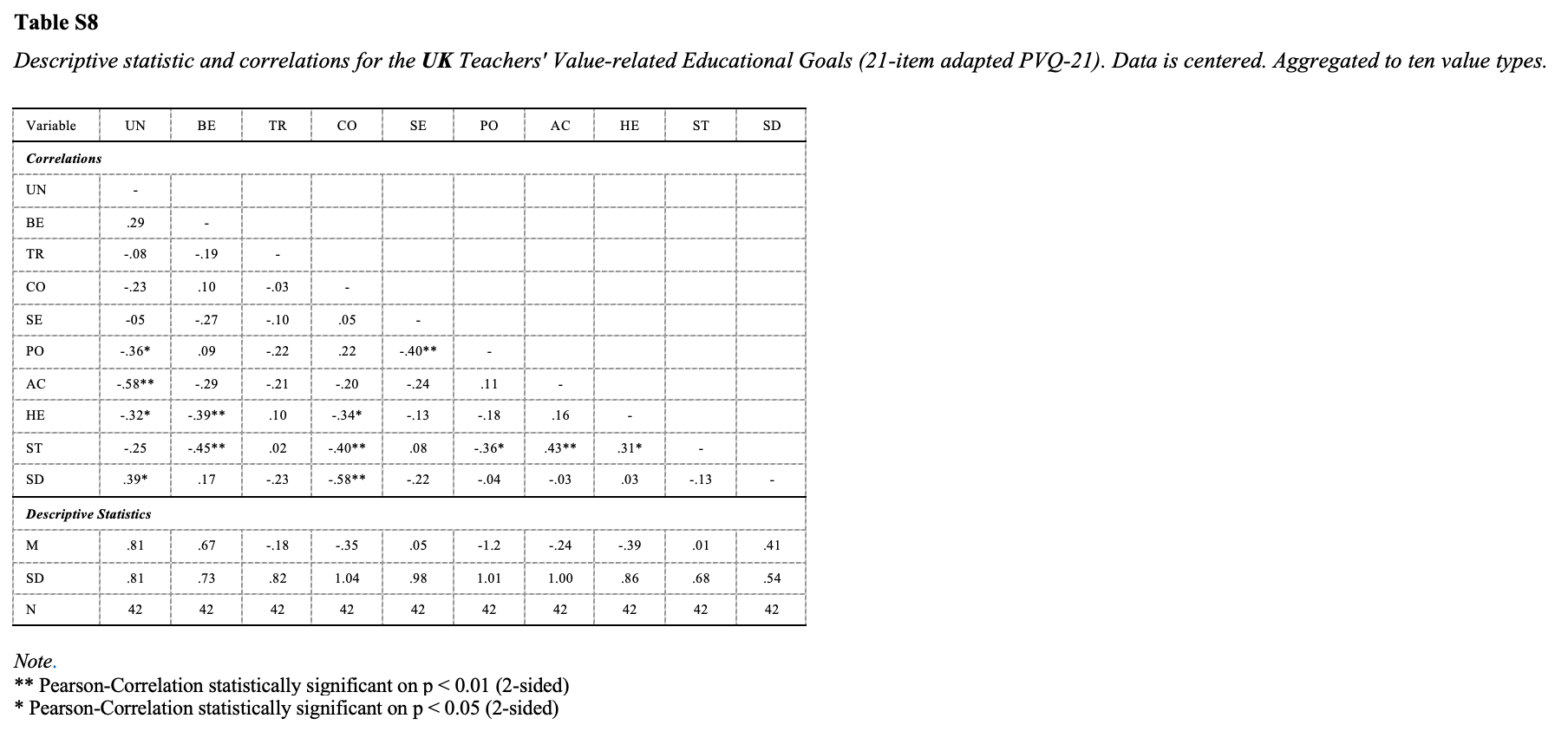

Supplement: Supplementary file 9 [file Image_8.png]

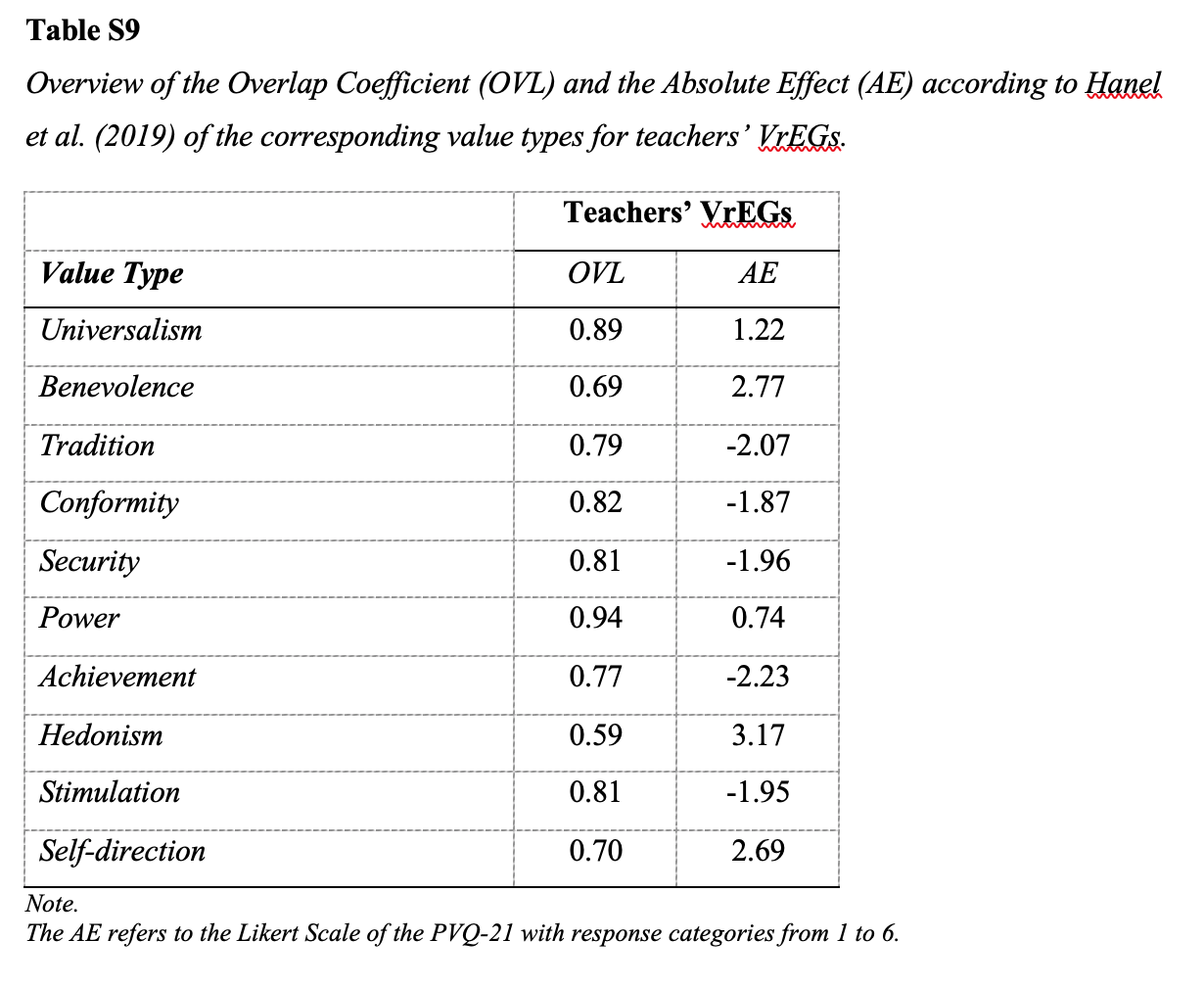

Supplement: Supplementary file 10 [file Image_9.png]
